# Increased levels of conjugated bile acids are associated with human bile reflux gastritis

**DOI:** 10.1038/s41598-020-68393-5

**Published:** 2020-07-14

**Authors:** Aihua Zhao, Shouli Wang, Wenlian Chen, Xiaojiao Zheng, Fengjie Huang, Xiaolong Han, Kun Ge, Cynthia Rajani, Yanxia Huang, Herbert Yu, Jinshui Zhu, Wei Jia

**Affiliations:** 1 Center for Translational Medicine and Shanghai Key Laboratory of Diabetes Mellitus, Shanghai Jiao Tong University Affiliated Sixth People’s Hospital, Shanghai, 200233 China; 2 University of Hawaii Cancer Center, Honolulu, 96813 USA; 3 Department of Gastroenterology, Shanghai Jiao Tong University Affiliated Sixth People’s Hospital, Shanghai, 200233 China

**Keywords:** Homeostasis, Biochemical networks

## Abstract

Bile acids (BAs) play essential roles in facilitating lipid digestion and absorption in the intestine. Gastric BAs were attributed to abnormal refluxing from duodenal compartments and correlated with the occurrence of gastric inflammation and carcinogenesis. However, the differences in gastric BAs between physiologically compromised and healthy individuals have not been fully investigated. In this study, gastric juice was collected from patients clinically diagnosed as gastritis with/without bile reflux and healthy subjects for BA profiles measurements. As a result, we found that the conjugated BAs became prominent components in bile reflux juice, whereas almost equal amounts of conjugated and unconjugated BAs existed in non-bile reflux and healthy juice. To investigate whether gastric BA changes were regulated by hepatic BA synthesis, C57BL/6J mice were intervened with GW4064/resin to decrease/increase hepatic BA synthesis. The results revealed that changes of gastric BAs were coordinated with hepatic BA changes. Additionally, gastric BAs were detected in several healthy mammals, in which there were no obvious differences between the conjugated and unconjugated BAs. Pigs were an exception. Thus, increased levels of conjugated BAs are associated with human bile reflux gastritis. Gastric conjugated BAs could become a panel of biomarkers to facilitate diagnosis of pathological bile reflux.

## Introduction

Bile acids (BAs) are a group of steroid acids with unique physical, chemical and biological characteristics that are one of the major components of bile^1–3^. Cholic acid (CA) and chenodeoxyocholic acid (CDCA) in human and α/β-muricholic acid (α/βMCA) in rodents are synthesized in hepatocytes from cholesterol. These primary BAs are conjugated with glycine or taurine to form the primary conjugated BAs that predominantly consist of glyco-CA (GCA), glyco-CDCA (GCDCA) in humans and tauro-CA (TCA), tauro-CDCA (TCDCA), tauro-α/βMCA (Tα/βMCA) in rodents. They are then secreted into bile canaliculi and stored in the gall bladder^1,4,5^. During a meal, bile fluid flows into the intestinal duct, where bile acids emulsify and solubilize lipid-soluble nutrients to facilitate dietary digestion and absorption. Meanwhile, primary BAs are metabolized by intestinal bacterial enzymes in deconjugation, dehydroxylation, oxidation or epimerization reactions to form a series of secondary BAs^6,7^. The majority of intestinal BAs are reabsorbed from the terminal ileum and transported back to the liver via the portal vein, to complete physiological, enteric circulation. Therefore, liver, gall bladder, and intestine are generally considered the main organs containing huge amount of BAs. However, some reports showed that high levels of BAs were present in the gastric juice of gastric ulcer, post-gastric surgery and normal subjects^8–10^. Thus, there may be a correlation between gastric BA composition and gastric disease. Until now, there have been few studies comparing gastric BA profiles between pathologically refluxing patients and healthy controls.

In order to explore characteristics of gastric BA, we collected human gastric juice from patients clinically diagnosed as having gastritis with bile reflux, as well as juice from gastritis without bile reflux and healthy subjects. BA profiles were analyzed in these samples and the results showed that BAs existed in both reflux and non-reflux gastric juice. Here, we intend to present the distinct BA profiles of bile reflux gastritis, to compare the similarities and differences of BA compositions between bile reflux juice and non-bile reflux juice. Meanwhile, we collected gastric content and gastric tissues from several healthy mammalian species to explore the characters of gastric BAs in the normal physiological state of normal mammals.

## Results

### BA profiles of human gastric juice

**Figure 1:**
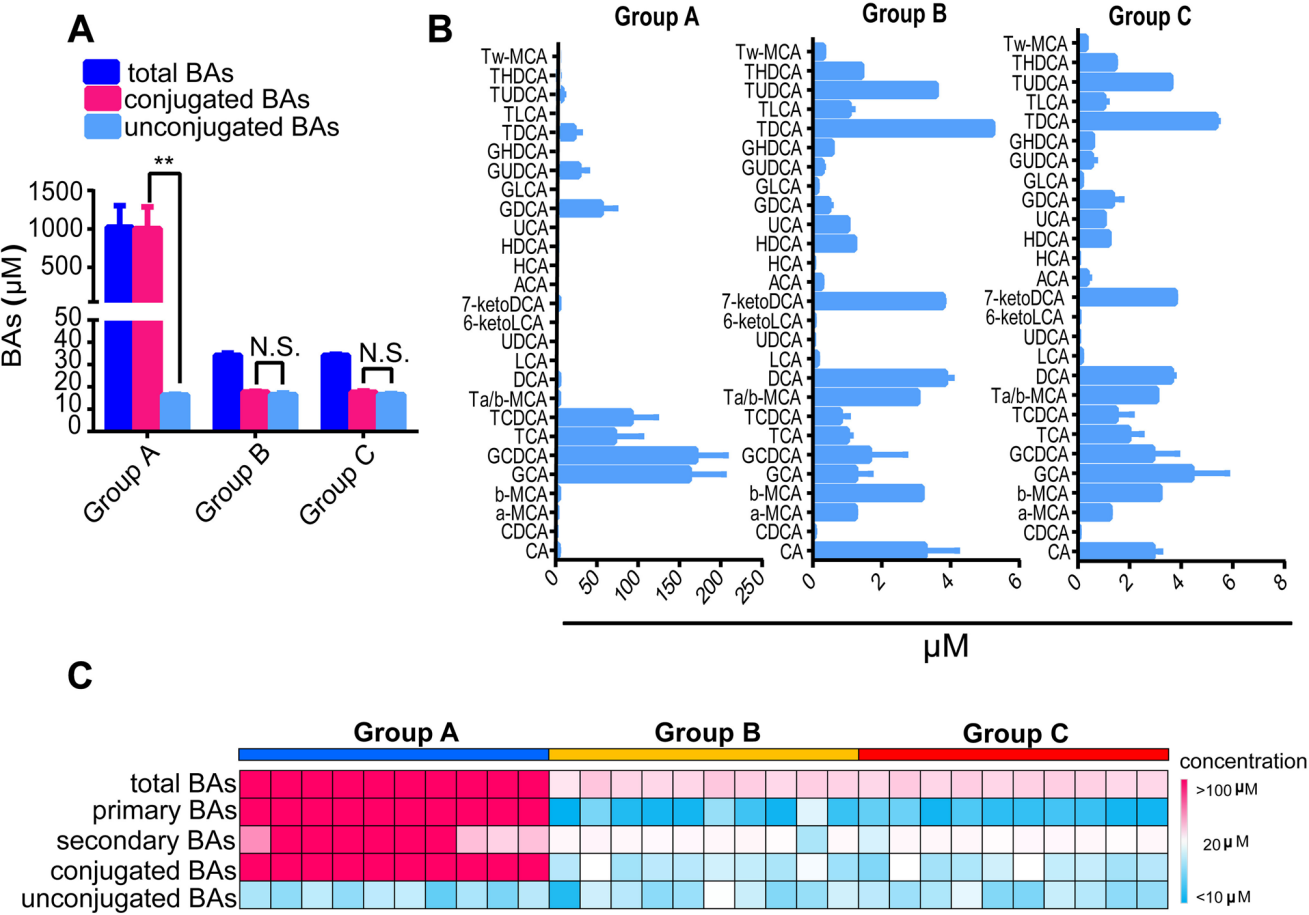
BA levels and compositions in human gastric juice. (**A**) The levels of total BAs, conjugated/unconjugated BAs in bile reflux juice (Group A, n = 10, non-bile reflux juice (Group B, n = 10) and healthy control (Group C, n = 10). Data expressed in mean ± SEM, **P < 0.01 when comparing conjugated and unconjugated BAs based on the student’s t-test. (**B**) The BA profiles of gastric juice from groups A, B and C. (**C**) The heatmap of total, primary/secondary, conjugated/unconjugated BAs in gastric juice from groups A, B and C. Each column represents one subject while each row represents one type of BAs. The color of one cell represents the concentration of one type BAs in each subject, in which darker red means higher concentration and lighter blue means lower concentration.

In order to explore the BA characteristics of human gastric juice, we measured the BA profiles of gastric juice from patients diagnosed with gastritis with bile reflux (group A), patients diagnosed with gastritis without bile reflux (group B) and control individuals without gastritis (group C). We found that there were high levels of BAs in the gastric juice from group A, reaching more than 1 mM (Fig. 1A) while there were less than 40 μM in both groups B and C. Although the 27 BA concentrations measured in gastric juice were much lower than those found in group A, all measured BA species in A could be detected in groups B and C (Fig. 1B). The percentage of conjugated BAs in group A was 98.4%, and significantly higher (*p* < 0.01) than those of unconjugated BAs, whereas the percentages of conjugated BAs in groups B and C were 51.5% and 51.7%, respectively, with no significant differences in unconjugated BA percentages ( *p* > 0.05). Additionally, we found that the profiles and concentrations of BAs, including primary and secondary BAs, conjugated and unconjugated BAs, in groups B and C were very similar (Fig. 1C, Table 1). In the Fig. 1C heatmap, the color of one cell represents the concentration of one type of BA in each subject, in which darker red means higher concentration and lighter blue means lower concentration. Of note, the levels of unconjugated BAs were quite similar among all three groups (Fig. 1A–C), suggesting that bile refluxing brings mainly conjugated BAs, such as GCA, GCDCA,TCA, TCDCA, into the stomach under pathological conditions, whereas almost equal amounts of conjugated and unconjugated BAs were distributed in gastric juice when obviously pathological bile reflux was absent.

**Table 1:** BA levels in gastric juice from bile reflux, non-bile reflux and healthy control (μM).

BAs	Bile flux	Non-bile reflux	Healthy control
	(Group A, n = 10)	(Group B, n = 10)	(Group C, n = 10)
CA	2.56 ± 1.04	2.99 ± 1.56	2.38 ± 1.09
CDCA	0.12 ± 0.11	0.03 ± 0.02	0.03 ± 0.04
a-MCA	1.23 ± 0.003	1.23 ± 0.01	1.23 ± 0.01
b-MCA	3.18 ± 0.01	3.17 ± 0.002	3.17 ± 0.02
GCA	266.03 ± 223.47	0.75 ± 0.82	0.74 ± 0.65
GCDCA	279.35 ± 198.07	0.82 ± 1.52	0.55 ± 0.50
TCA	126.14 ± 196.16	0.83 ± 0.06	0.87 ± 0.10
TCDCA	157.49 ± 171.9	0.58 ± 0.21	0.57 ± 0.08
Ta/b-MCA	3.30 ± 0.75	3.04 ± 0.002	3.05 ± 0.003
DCA	3.58 ± 0.4	3.75 ± 0.39	3.78 ± 0.60
LCA	0.12 ± 0.03	0.13 ± 0.02	0.12 ± 0.02
UDCA	0.03 ± 0.02	0.01 ± 0.01	0.02 ± 0.02
6-ketoLCA	0.02 ± 0.01	0.03 ± 0.01	0.02 ± 0.01
7-ketoDCA	3.76 ± 0.03	3.77 ± 0.02	3.75 ± 0.02
ACA	0.26 ± 0.02	0.25 ± 0.01	0.25 ± 0.01
HCA	0.01 ± 0.01	0.02 ± 0.01	0.01 ± 0.01
HDCA	1.21 ± 0.03	1.20 ± 0.02	1.21 ± 0.02
UCA	1.02 ± 0.01	1.01 ± 0.01	1.01 ± 0.01
GDCA	84.51 ± 94.95	0.37 ± 0.16	0.39 ± 0.20
GLCA	1.93 ± 3.38	0.12 ± 0.004	0.12 ± 0.007
GUDCA	45.13 ± 62.31	0.26 ± 0.14	0.24 ± 0.08
GHDCA	0.57 ± 0.03	0.56 ± 0.0005	0.55 ± 0.001
TDCA	38.09 ± 37.77	5.21 ± 0.003	5.22 ± 0.02
TLCA	n.d	0.93 ± 0.13	0.86 ± 0.01
TUDCA	10.07 ± 13.46	3.58 ± 0.002	3.58 ± 0.002
THDCA	4.16 ± 5.42	1.42 ± 0.0007	1.41 ± 0.001
Tw-MCA	0.32 ± 0.03	0.30 ± 0.01	0.31 ± 0.01

Data expressed as mean ± SEM; *n.d.* not detected.

We further measured the pH of the gastric juice and results were 4.62 ± 2.20, 2.73 ± 0.90, 2.37 ± 0.94, respectively in groups A to C. Moreover, the total BA levels of the three groups were positively correlated (r = 0.363, *p* < 0.05) with the pH of gastric juice. The conjugated BAs were significantly and positively correlated (r = 0.494, *p* < 0.01) with the pH whereas, the unconjugated BAs were not correlated (*p* = 0.595) with pH, implying that increasing gastric pH accompanies increasing amounts of BAs, especially reflux-induced conjugated BAs.

### The levels of BAs in gastric tissues are comparable to BA levels in hepatic tissues in C57BL/6J mice

**Figure 2:**
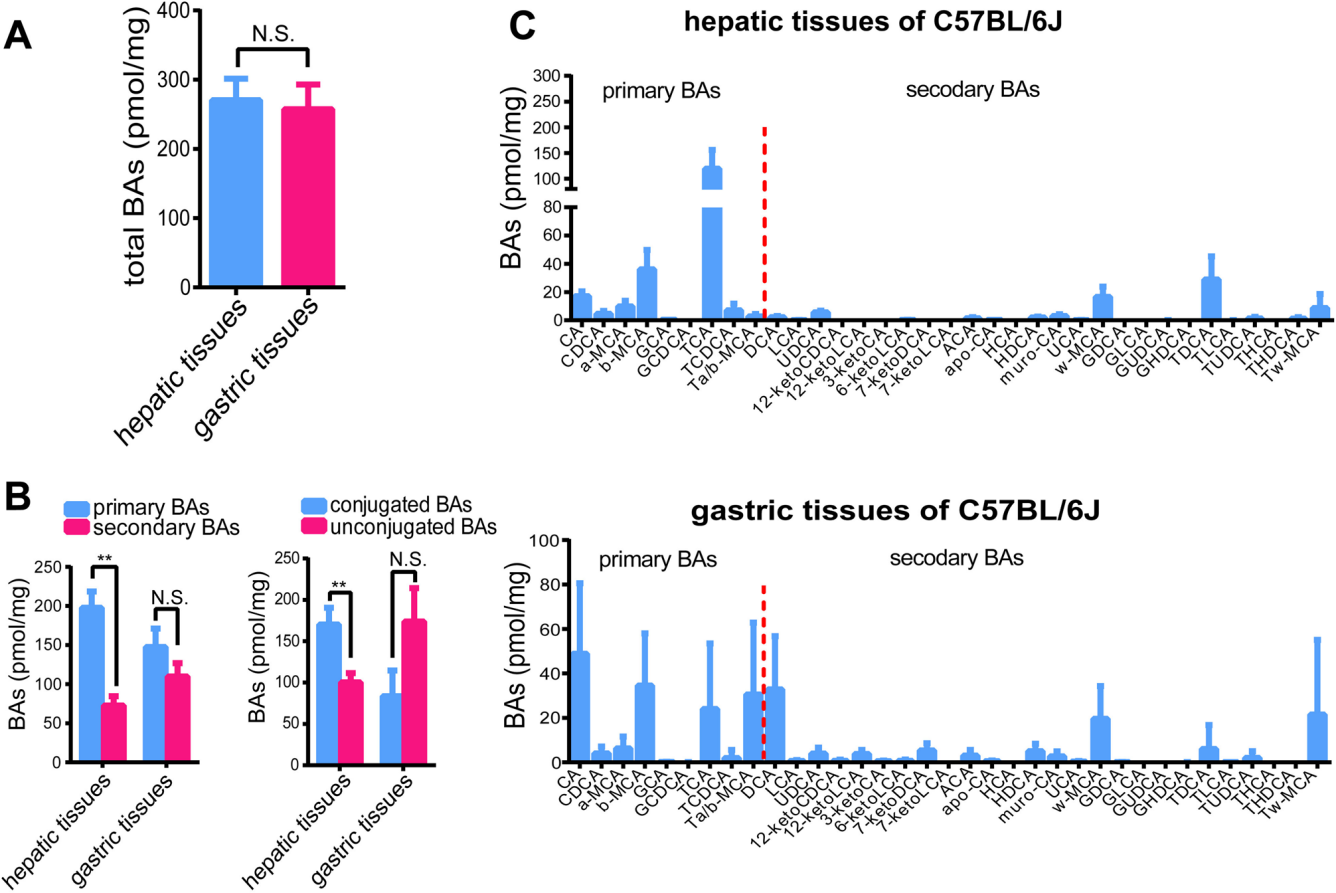
Comparison of BA levels and composition between hepatic tissues and gastric tissues of C57BL/6J mice. (**A**) Total primary/secondary and conjugated/unconjugated BA levels between hepatic and gastric tissues of C57BL/6J mice (n = 8). (**B**) Primary/secondary, conjugated/unconjugated BA levels within the hepatic tissues and gastric tissues, respectively. Data expressed mean ± SEM, **P < 0.01 based on the unpaired student’s t-test. (**C**) BA compositions of hepatic tissues and gastric tissues. The left bars (red dotted line) represent primary BAs and the right bars (red dotted line) represent secondary BAs.

Clinical results were consistent with previous reports that BAs were distributed in healthy stomachs^9,10^, and suggested that the characteristics of almost equal amounts of conjugated and unconjugated BAs corresponded to no obviously pathological bile refluxing conditions. In order to investigate the physiological gastric BA levels and composition further, we collected gastric tissues from healthy C57BL/6J mice, as well as their hepatic tissues, the organ where the BAs are synthesized, to measure and compare their BA levels. High concentrations of total BAs (257.5 pmol/mg) were quantified in gastric tissues, with results that were very close to the level found in the corresponding hepatic tissues (270.5 pmol/mg) (*p* > 0.05) (Fig. 2A). However, the levels of primary and secondary BAs, conjugated and unconjugated BAs within hepatic tissues were significantly different from each other (*p* < 0.01), but there were no significant differences observed within gastric tissues (Fig. 2B). Thus, although the BA levels of gastric tissues were comparable to those in the hepatic tissues, the profiles and compositions of BA between gastric and hepatic tissues were quite different (Fig. 2C). Moreover, the results for gastric BA composed almost amounts of conjugated and unconjugated BAs under normal physiological conditions in mice were consistent with those observed in humans.

### Gastric BAs correlated with hepatic BAs in healthy C57BL/6J mice

**Figure 3:**
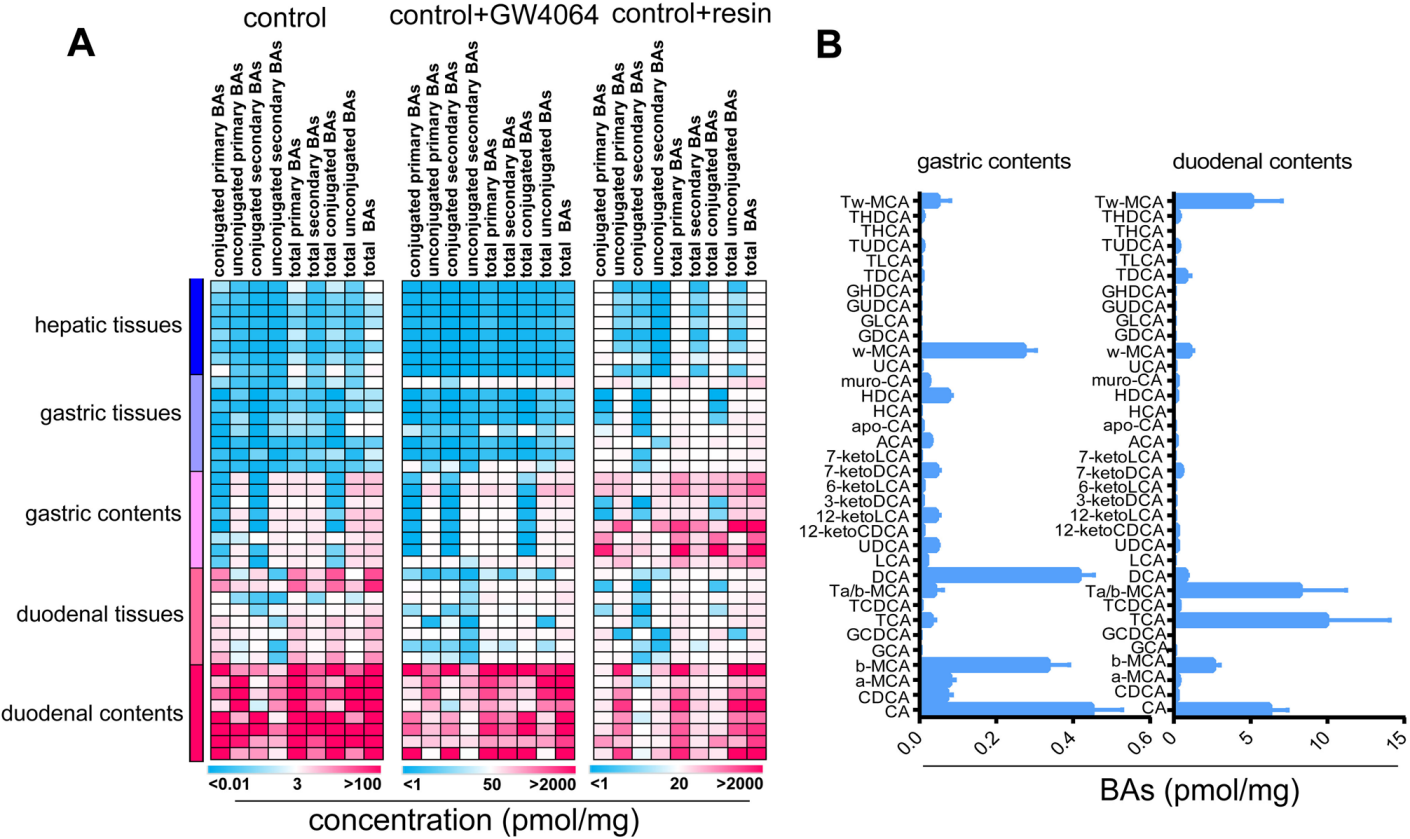
The BA compositions among gastric tissues, gastric contents, duodenal tissues and duodenal contents of C57BL/6J mice. (**A**) The heatmap of hepatic/gastric/deudenal tissues and gastric/duodenal contents of C57BL/6J mice with GW4064 or resin intervention (n = 8). Each column represents one type of BAs while each row represents one mouse. The color of one cell represents the concentration of one type of BAs in a mouse, in which darker red means higher concentration and lighter blue means lower concentration. (**B**) BA compositions of gastric tissues and gastric contents of C57BL/6J mice.

After food intake, BAs stored in the gall bladder are secreted into the duodenal compartment resulting in high levels of BAs. Previously, the BAs found in gastric juice were hypothesized to reflux from the duodenal compartment under pathological conditions. To further elucidate whether gastric BA composition was correlated with hepatic BA composition, we compared BA changes in gastric contents/tissues, duodenal contents/tissues and hepatic tissues in C57BL/6J mice. First, we carried out the comparison of primary/secondary BAs, conjugated/unconjugated BAs and total BAs under normal physiological condition. As previously mentioned, BA levels in gastric tissues were very close to those in hepatic tissues, whereas BA levels were observed to gradually increase in gastric content, duodenal tissues and duodenal contents, implying that gastric BAs most likely came from duodenal refluxing (Fig. 3A). In the heatmaps, the color of one cell represents the concentration of one type of BAs in a mouse, in which darker red means higher concentration and lighter blue means lower concentration. We then used GW4064, a Farnesoid X receptor (FXR) agonist to suppress hepatic BA synthesis and reduce BA pools^7,11^, resulting in decreased levels and changed composition of BAs in hepatic tissues. The levels of BA in gastric tissues were consistent with the changes observed in hepatic tissues, while BA levels were also reduced to some degree in gastric contents, duodenal tissues and contents, compared to those that were not treated with GW4064, although gradual increases in gastric BAs were observed over time. Lastly, the mice were treated with resin, which acting as a BA sequestrate, bound to and cleared intestinal BAs resulting in an increase in hepatic BA synthesis^12,13^. Our results showed that BAs in gastric tissues, contents and hepatic tissues were increased when intestinal BAs were cleared by resin, further implying that gastric BA changes were consistent with hepatic BAs. Although we found that BA levels in gastric contents and duodenal contents were very different, the BA compositions were very similar (Fig. 3B), providing supporting evidence that gastric BAs come from duodenal refluxing in the normal mice.

### The BA profiles of gastric tissues in different mammals

**Figure 4:**
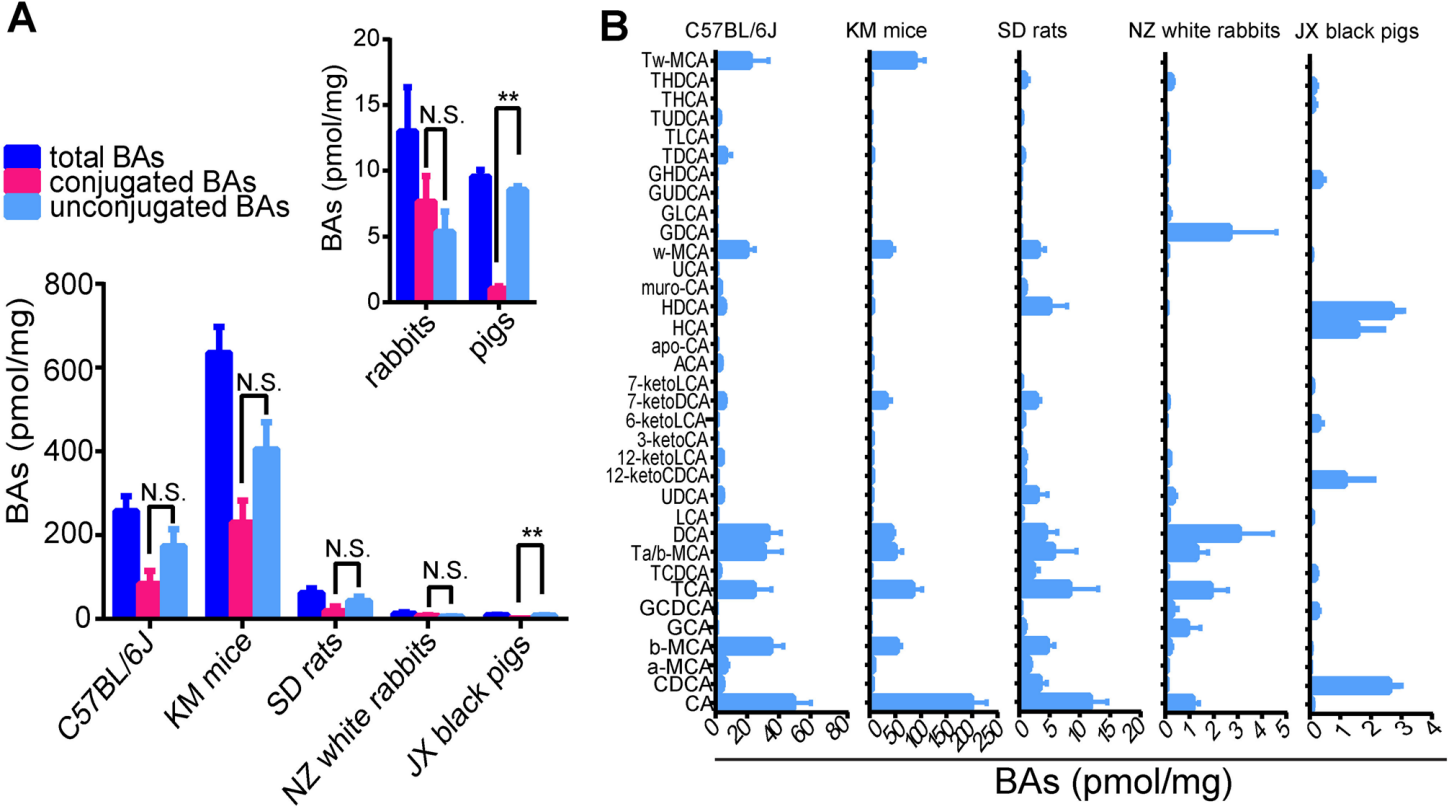
The BA levels and profiles in mammalian animal gastric tissues. (**A**) The levels of total, conjugated/unconjugated BAs in the gastric tissues of C57BL/6J mice (n = 8), KM mice (n = 6), SD rats (n = 6), NZ white rabbits (n = 4) and JX black pigs (n = 3). Data expressed in mean ± SEM, **P < 0.01 when comparing conjugated BAs and unconjugated BAs based on the unpaired student’s t-test. (**B**) The BA compositions among gastric tissues from C57BL/6J mice, KM mice, SD rats, NZ white rabbits and JX black pigs.

To further explore the features of gastric BAs, we collected gastric tissues from healthy KM mice, SD rats, NZ white rabbits, and JX black pigs, and measured BA levels and compositions in these samples. The results showed that the total BA levels of rats, rabbits and pigs were relatively lower than those in mice (Table 2, Fig. 4A). Except for JX black pigs, there were no significant variations between the levels of conjugated and unconjugated BAs in these mammalian gastric tissues under normal physiological conditions, consistent with the results observed from clinically diagnosed non-refluxing gastritis and healthy human subjects. Furthermore, CA was the major component in primary BAs of mice, SD rats and NZ white rabbits, whereas CDCA was the more prominent compound in JX black pigs (Fig. 4B). The percentages of primary BAs, including CA, CDCA, αMCA, βMCA, TCA, TCDCA, TαMCA, TβMCA, GCA and GCDCA, were 57.04 ± 0.10%, 62.26 ± 0.05%, 61.0 ± 0.20%, 50.66 ± 0.12% in C57BL/6J mice, KM mice, SD rats and NZ white rabbits, respectively, whereas there was only 32.45 ± 0.02% found in JX black pigs. Another characteristic of BA profiles for JX black pigs was that the concentrations of hyocholic acid (HCA) and hyodeoxyhcholic acid (HDCA) were the highest of all detected BAs except CDCA, the sum of these two BAs percentages was 43.81 ± 0.09%, implying that pigs have a unique gastric BA panel from the other tested mammalian species.

**Table 2:** BA levels of hepatic tissues from C57BL/6J mice and gastric tissues of C57BL/6J, KM mice, SD rats, NZ white rabbits and JX black pigs (pmol/mg).

Bile acid	Hepatic tissues of C57BL/6J mice (n = 8)	Gastric tissues of C57BL/6J mice (n = 8)	Gastric tissues of KM mice (n = 8)	Gastric tissues of SD rats (n = 6)	Gastric tissues of NZ white rabbits (n = 4)	Gastric issues of JX black pigs (n = 3)
CA	17.15 ± 3.38	48.91 ± 31.68	198.15 ± 74.62	11.69 ± 6.87	1.13 ± 0.52	0.07 ± 0.02
CDCA	4.73 ± 1.92	4.05 ± 3.04	3.95 ± 2.57	3.35 ± 2.99	0.05 ± 0.03	2.60 ± 0.37
a-MCA	9.94 ± 4.02	6.28 ± 5.35	7.15 ± 4.76	1.60 ± 0.87	0.06 ± 0.03	n.d
b-MCA	36.16 ± 13.69	34.57 ± 23.48	53.00 ± 20.19	4.55 ± 3.04	0.19 ± 0.21	0.02 ± 0.003
GCA	0.44 ± 0.04	0.21 ± 0.12	0.18 ± 0.10	0.70 ± 0.91	0.90 ± 1.10	
GCDCA	n.d	0.05 ± 0.04	0.14 ± 0.03	0.16 ± 0.14	0.29 ± 0.43	0.24 ± 0.05
TCA	119.31 ± 36.64	24.07 ± 29.48	83.26 ± 46.49	8.26 ± 11.78	1.88 ± 1.44	n.d
TCDCA	7.18 ± 4.65	1.97 ± 3.66	1.60 ± 0.52	2.26 ± 2.34	0.07 ± 0.03	0.18 ± 0.03
Ta/b-MCA	2.8 ± 1.72	30.53 ± 32.31	48.53 ± 36.41	5.56 ± 9.51	1.29 ± 0.96	n.d
DCA	2.17 ± 0.87	32.86 ± 23.91	42.14 ± 14.73	4.23 ± 4.93	3.03 ± 2.82	n.d
LCA	0.22 ± 0.10	0.62 ± 0.42	1.49 ± 0.77	0.39 ± 0.29	0.10 ± 0.07	0.07 ± 0.01
UDCA	5.64 ± 1.26	3.94 ± 2.70	3.48 ± 0.95	2.86 ± 4.4	0.32 ± 0.27	n.d
12-ketoCDCA	n.d	0.75 ± 0.50	4.46 ± 2.49	0.76 ± 0.51	0.05 ± 0.03	1.14 ± 0.95
12-ketoLCA	n.d	3.82 ± 1.79	3.54 ± 2.32	0.80 ± 0.91	0.17 ± 0.10	n.d
3-ketoCA	n.d	0.5 ± 0.32	4.10 ± 4.57	0.10 ± 0.02	n.d	n.d
6-ketoLCA	0.34 ± 0.09	0.73 ± 0.53	0.93 ± 0.45	0.64 ± 0.37	0.01 ± 0.005	0.28 ± 0.12
7-ketoDCA	n.d	5.41 ± 3.19	31.14 ± 26.12	2.74 ± 1.83	0.10 ± 0.10	n.d
7-ketoLCA	n.d	n.d	0.82 ± 0.34	0.28 ± 0.13	n.d	0.08 ± 0.01
ACA	1.52 ± 0.78	3.06 ± 2.54	3.54 ± 2.43	n.d	n.d	n.d
apo-CA	0.33 ± 0.07	0.5 ± 0.36	0.47 ± 0.19	n.d	n.d	n.d
HCA	n.d	n.d	n.d	n.d	n.d	1.56 ± 0.85
HDCA	2.02 ± 0.6	5.06 ± 3.28	5.16 ± 3.24	4.97 ± 7.25	0.05 ± 0.01	2.68 ± 0.37
muro-CA	3.16 ± 1.24	2.71 ± 2.28	1.62 ± 1.43	0.84 ± 0.71	n.d	n.d
UCA	0.22 ± 0.09	0.39 ± 0.21	0.36 ± 0.16	0.07 ± 0.04	0.01 ± 0.02	n.d
w-MCA	16.66 ± 7.13	19.64 ± 14.74	39.5 ± 21.6	3.08 ± 2.89	0.07 ± 0.09	0.04 ± 0.01
GDCA	n.d	0.09 ± 0.10	0.12 ± 0.02	0.1 ± 0.04	2.65 ± 3.88	n.d
GLCA	n.d	0.02 ± 0.002	0.01 ± 0.001	0.01 ± 0.001	0.12 ± 0.20	n.d
GUDCA	0.09 ± 0.06	0.02 ± 0.01	0.04 ± 0.02	0.03 ± 0.02	0.02 ± 0.01	n.d
GHDCA	n.d	0.06 ± 0.02	0.07 ± 0.03	0.15 ± 0.12	0.01 ± 0.01	0.34 ± 0.11
TDCA	28.9 ± 16.44	6.04 ± 10.79	5.26 ± 4.10	0.52 ± 0.77	0.11 ± 0.03	n.d
TLCA	0.1 ± 0.05	0.09 ± 0.04	0.08 ± 0.02	n.d	0.01 ± 0.004	n.d
TUDCA	1.42 ± 1.15	2.03 ± 0.30	1.16 ± 0.64	0.33 ± 0.46	0.04 ± 0.03	n.d
THCA	n.d	n.d	n.d	n.d	n.d	0.11 ± 0.07
THDCA	1.25 ± 0.98	n.d	1.69 ± 0.72	0.97 ± 1.43	0.26 ± 0.19	0.15 ± 0.06
Tw-MCA	8.76 ± 9.84	21.53 ± 33.58	87.96 ± 48.96	n.d	n.d	n.d

Data expressed as mean ± SEM; n.d.: not detected.

## Discussion

BAs have well-established roles in facilitating fat digestion and absorption, are also important signaling molecules regulating cholesterol homeostasis and glucose, lipid metabolism^1,3^. Ordinarily, BAs exist mainly in enterohepatic organs where they maintain their physiological circulation, thus liver, gall bladder and intestine contain high levels of BAs. Stomach is an important digestive organ which is not involved in BA enterohepatic circulation and would not be expected to contain high levels of BAs. However, considerable amounts of duodenal-gastric BA reflux have been detected in normal subjects and in post-gastric surgery patients without symptoms^8,14^. Our results validated that a substantial amount of BAs are distributed in the normal gastric juice and in the gastric juice of patients with gastritis with non-bile reflux, as well as in the gastric tissues and contents from several healthy mammalian species. These results demonstrated that duodenal-gastric refluxing exists under both physiological and pathological conditions in humans, and that the physiological roles of gastric BAs need to be further investigated. As we observed in the human and animal experiments, there were almost equal levels of conjugated and unconjugated BAs in normal gastric tissues, suggesting that there likely exists a different model for bile reflux between normally physiological and abnormally pathological conditions. BA profiles were markedly different in gastritis patients with bile reflux and were characterized by increased amounts of conjugated BAs in the stomach, as well as in the pathological refluxing juice. Thus, we speculated that the ratio of conjugated BAs to total BAs is a crucial factor for discrimination between physiological and pathological refluxing. Moreover, when an amount of conjugated BAs are refluxed into stomach, the strong acidic gastric environments were perturbed. We observed that gastric juice pH values of gastritis patients with clinical bile refluxing were increased to 4.62 from values of 2.73 and 2.37 that were found in gastritis patients with no obvious bile refluxing and healthy subjects. We hypothesize that this may result in additional injury to the gastric mucosa which in turn, may lead to increased pathological changes.

Recent studies revealed that high level of BAs in human gastric juice contribute to the progression of histological atrophy and intestinal metaplasia, followed by gastric carcinogenesis in patients^15^. The role of BAs in initiating and promoting cancer of the digestive system including liver cancer and colorectal cancer, has been previously highlighted^16–18^. In this study, we found that gastritis patients with bile reflux had BA levels that were significantly increased relative to those subjects with gastritis with no bile reflux and non-gastritis, especially with respect to conjugated BAs. Therefore, the increased conjugated BAs in gastric tissues caused by refluxing could be major risk factors to increase the developing of various gastric diseases. According to previous reports, the mixture of GCA, GCDCA, GDC, TCA, TCDCA and TDCA which was very similar to the prominent components in bile refluxing juice, were able to induce epithelial to mesenchymal transition via VEGF signaling in non-neoplastic Barrett’s cells in vitro^19^. Long term exposure to high levels of conjugated BAs, such as GCDCA, has been shown to induce oxidative DNA damage and promote carcinogenesis in the biliary tract^20^. Our results provided supporting evidence that increased gastric conjugated BAs were a distinguishing factor for gastritis patients with bile reflux. Additionally, our studies provided details of gastric BA profiles in human and several mammals with the finding that, under physiological conditions, they have similar ratios of conjugated to unconjugated BAs with the exception of JX black pigs. Thus, the elevation of gastric conjugated BAs may potentially be used as a biomarker to facilitate diagnosis of pathological bile refluxing.

In this paper, we have performed a comprehensive evaluation of gastric BAs involving clinical samples and several male animals using a metabolomic approach. However, this is an observational study, further studies are warranted to fully define mechanistic roles of conjugated BAs in the development of gastritis, gastric cancer, as well as the physiological role of gastric BAs. Also, we will carry out these investigations in female mice.

## Methods

### Human sample collection

A total of 30 subjects were enrolled in this study between August and September 2017, including age- and gender- matched patients diagnosed with gastritis with bile reflux (n = 10, group A), patients diagnosed with gastritis without bile reflux (n = 10, group B) and control individuals without gastritis (n = 10, group C). All study subjects underwent a routine gastroscopic examination in the Shanghai Jiao Tong University Affiliated Sixth People’s Hospital, and the diagnosis of gastritis was made according to the Chinese national consensus on chronic gastritis. Bile reflux was diagnosed by seeing yellow-green liquid in gastroscopic view and a pH value higher than 4. Patients who were receiving treatment for *Helicobacter pylori* infection or taking proton pump inhibitors or had previous gastrectomy were excluded from the study. About 10 ~ 15 mL of gastric juice was collected from the gastric corpus of each participant during the endoscopic examination. The biological specimens were placed in dry ice immediately after collection, and then stored in a − 80 °C freezer until analysis.

The sample collection and study was approved by the institutional human subjects review board of Shanghai Jiao Tong University Affiliated Sixth People’s Hospital, Shanghai, China. All participants signed informed consent forms prior to the study. All methods were carried out in accordance with the approved guidelines.

### Animal experiments

All animal experiments were performed in accordance with the Guide for the Care and Use of Laboratory Animals of the National Institutes of Health. The experimental protocols were approved by the Center for Laboratory Animals, Shanghai Jiao Tong University Affiliated Sixth People’s Hospital, Shanghai, China.

All animals were obtained from the Shanghai Laboratory Animal Co. LTD. (SLAC, Shanghai, China). Animals were raised in specific pathogen free environment under 20–22 °C and 45 ± 5% humidity with a 12 h light/12 h dark cycle with access to ultrapure water and standard chow ad libitum. They were acclimatized for one week before experiments.

A total of 24 four-week-old male C57BL/6J mice were divided into 3 groups (8 per group): (1) control group fed with a normal chow (control group); (2) GW4064 intervention group fed with a standard chow accompanied with a dose of 180 mg/kg (wt/wt) GW4064 for 8 weeks (control + GW4064 group); and (3) Resin intervention group fed with 2% cholestyramine resin (wt/wt) in standard chow for 8 weeks (control + resin group). At the end of the experiments, all of the mice were sacrificed after 12 h fasting. The mice were placed in sterilized cages with new pads before fasting, and were given free access to water during fasting. The hepatic tissues, gastric tissues/contents and duodenal tissues/contents were collected after sacrifice. Whole stomach/duodenal resections were slightly scrapped to collect gastric/duodenal contents, which were mixtures of chyme and mucosal juice. The stomach/duodenal resections, which were regarded as gastric/duodenal tissues, were washed with phosphate buffer saline (PBS) to remove the residual blood and other contaminants. All of the collected samples were snap frozen in liquid nitrogen immediately after collection, and then moved to a − 80 °C freezer until analysis.

Six four-week-old male Kunming (KM) mice, 6 four-week-old male Sprague–Dawley (SD) rats, and 4 six-month-old male New Zealand (NZ) white rabbits were also used in this experiment. The animals were fasted for 12 h, and then sacrificed to collect gastric tissues and contents. All obtained gastric tissues were washed with PBS, kept temporarily in liquid nitrogen and stored at − 80 °C until analysis. The gastric tissues of male three 3-month-old Jiaxing (JX) black pigs were washed with PBS after removing contents, and then frozen immediately in liquid nitrogen before moving to a − 80 °C freezer for storage.

### BA quantification

All bile acid standards: including cholic acid (CA), chenodeoxycholic acid (CDCA), α-muricholic acid (α-MCA), β-muricholic acid (β-MCA), taurocholic acid (TCA), taurochendeoxycholic acid (TCDCA), tauro α-muricholic acid (Tα-MCA), tauro β-muricholic acid (Tβ-MCA), glycocholic acid (GCA), glycochenodeoxycholic acid (GCDCA), deoxycholic acid (DCA), lithocholic acid (LCA), ursodeoxycholic acids (UDCA), 12-keto chenodeoxycholic acid (12-ketoCDCA), 12-keto lithocholic acid (12-ketoLCA), 3-keto cholic acid (3-ketoCA), 6-keto lithocholic acid (6-ketoLCA), 7-keto deoxycholic acid (7-ketoDCA), 7-keto lithocholic acid (7-ketoLCA), allo-cholic acid (ACA), apocholic acid (apoCA), hyocholic acid (HCA), hyodeoxycholic acid (HDCA), murocholic acid (muro-CA), ursocholic acid (UCA), ω-muricholic acid (ω-MCA), glycodeoxycholic acid (GDCA), glycolithocholic acid (GLCA), glycoursodeoxycholic acid (GUDCA), glycohyodeoxycholic acid (GHDCA), taurodeoxycholic acid (TDCA), taurolithocholic acid (TLCA), tauroursodeoxycholic acid (TUDCA), taurohyodeoxycholic acid (THDCA), tauro ω-muricholic acid (Tω-MCA), cholic acid-d4 (CA-d4), lithocholic acid-2,2,4,4-d4 (LCA-d4), ursodeoxycholic acid-2,2,4,4-d4 (UDCA-d4), glycocholic acid-2,2,4,4-d4 (GCA-d4), glycochenodeoxycholic acid-2,2,4,4-d4 (GCDCA-d4) and glycodeoxycholic acid-2,2,4,4-d4 (GDCA-d4), were obtained from Steraloids ( United States).

Sample preparation was based on previous protocols established by our lab with minor optimization^21,22^. Briefly, 50 μL of gastric juice were pipetted and then extracted with a 300 μL mixture of acetonitrile and methanol (8:2, v/v), containing 6 internal standards (IS), 50 nM each of CA-d4, UDCA-d4, LCA-d4, GCA-d4, GDCA-d4 and GCDCA-d4. After centrifugation at 13,000 rpm for 15 min, the supernatant was diluted 15-fold with extraction solvent. An aliquot of 60 µL diluent of each sample was used for bile acid quantification with standard curves. For tissues and contents quantification, gastric tissues/contents (about 10 mg), duodenum tissues/contents (about 10 mg) were homogenized for 5 min in a 200 µL mixture of methanol and water (1:1, v/v) containing the 6 IS described above. After centrifugation at 13,000 rpm for 15 min, the supernatant was transferred to a 1.5 mL tube. The residue was rehomogenized for 5 min in a 200 µL mixture of methanol and acetonitrile (2/8, v/v) containing IS. After centrifugation at 13,000 rpm for 15 min, the supernatant was removed and combined into one tube. Each combined supernatant was vortexed, and then centrifuged at 13,000 rpm for 10 min and the supernatant of each sample was used for quantification.

All samples were quantitatively measured using UPLC-TQMS (Waters Corp., Milford, MA). All separations were performed on a Waters ACQUITY BEH C18 column, 100 mm × 2.1 mm, 1.7 μm (Waters, Milford, MA). The mobile phase A consisted of 0.01% formic acid in Millipore water, and mobile phase B consisted of LC–MS grade acetonitrile and methanol (87:13 v/v). The flow rate was set at 0.45 mL/min. The mobile phase gradient was based on our previous protocol^21,22^. The injection volume of all tested samples was 5 µL. The mass spectrometer was run on negative mode with the source temperature at 150 °C and desolvation temperature at 550 °C, respectively. Data acquisition was performed using MassLynx version 4.1, and BA quantification was performed using the TargetLynx applications manager version 4.1 (Waters, Milford, MA).

### Statistical analysis

All data was analyzed using GraphPad Prism 6 (GraphPad Software, USA). Data was expressed as mean ± SEM. The significance of differences between groups was evaluated using the t-student’s test. *P* values less than 0.05 were considered as statistically significant. Heatmaps were generated in Microsoft excel 2010.
